# Network analysis of wildfire transmission and implications for risk governance

**DOI:** 10.1371/journal.pone.0172867

**Published:** 2017-03-03

**Authors:** Alan A. Ager, Cody R. Evers, Michelle A. Day, Haiganoush K. Preisler, Ana M. G. Barros, Max Nielsen-Pincus

**Affiliations:** 1 Missoula Fire Sciences Laboratory, Rocky Mountain Research Station, Forest Service, United States Department of Agriculture, Missoula, Montana, United States of America; 2 Department of Environmental Sciences and Management, Portland State University, Portland, Oregon, United States of America; 3 Department of Forest Ecosystems & Society, College of Forestry, Oregon State University, Corvallis, Oregon, United States of America; 4 Pacific Southwest Research Station, Forest Service, United States Department of Agriculture, Albany, California, United States of America; 5 Department of Forest Engineering, Resources & Management, College of Forestry, Oregon State University, Corvallis, Oregon, United States of America; University of Vermont, UNITED STATES

## Abstract

We characterized wildfire transmission and exposure within a matrix of large land tenures (federal, state, and private) surrounding 56 communities within a 3.3 million ha fire prone region of central Oregon US. Wildfire simulation and network analysis were used to quantify the exchange of fire among land tenures and communities and analyze the relative contributions of human versus natural ignitions to wildfire exposure. Among the land tenures examined, the area burned by incoming fires averaged 57% of the total burned area. Community exposure from incoming fires ignited on surrounding land tenures accounted for 67% of the total area burned. The number of land tenures contributing wildfire to individual communities and surrounding wildland urban interface (WUI) varied from 3 to 20. Community firesheds, i.e. the area where ignitions can spawn fires that can burn into the WUI, covered 40% of the landscape, and were 5.5 times larger than the combined area of the community core and WUI. For the major land tenures within the study area, the amount of incoming versus outgoing fire was relatively constant, with some exceptions. The study provides a multi-scale characterization of wildfire networks within a large, mixed tenure and fire prone landscape, and illustrates the connectivity of risk between communities and the surrounding wildlands. We use the findings to discuss how scale mismatches in local wildfire governance result from disconnected planning systems and disparate fire management objectives among the large landowners (federal, state, private) and local communities. Local and regional risk planning processes can adopt our concepts and methods to better define and map the scale of wildfire risk from large fire events and incorporate wildfire network and connectivity concepts into risk assessments.

## Introduction

As fire events in the western US and elsewhere have become increasingly large and destructive, developing long-term social and ecological strategies to mitigate impacts remains a challenge [1]. A wide range of solutions have been advocated in recent literature, including changing existing suppression policies to allow more natural ignitions so that wildfires eventually become self-limiting [2], improving the efficiency of programs to treat and reduce wildland fuels [3], changing management culture and attitudes towards fire [4], and incorporating new analytical tools for wildfire scenario planning to improve the coupling of human and biophysical subsystems [1, 5]. The wide ranging discussions reinforce the fact that managing long-term risk from large, highly uncertain wildfire events is a complex socioecological problem that will require rapid adjustments in existing risk governance systems in a changing climate. By risk governance, we mean the process by which authority is exercised and decisions are taken within social and institutional environments to identify, assess, manage, and communicate risk [6, 7]. Existing risk governance is challenged by the fine-grained diversity of land tenures and socioecological settings (e.g. fire regime, attitude towards fire) relative to the scale of large fire events [8, 9].

Perspectives on wildfire management are often deeply divisive among public and private entities, and a consistent perception of risk impedes the development of social organizations that are needed to align goals with respect to wildfires and their impacts [9, 10]. For instance, in the western US, state wildfire management agencies have a public mandate to protect private lands from wildfire, while federal policy on adjacent fire-adapted forests encourages increased use of prescribed and managed wildfire to reduce fuels and wildfire risk [11]. The increased use of wild and prescribed fire as a fuel management strategy on public forests [12] in particular has and will continue to generate fire management conflicts among state, federal, and private land jurisdictions. Another example is the management of private industrial timberlands, where the lack of financial incentives to remove surface fuels generated from harvesting can exacerbate wildfire risk to surrounding parcels [13–15]. At the community scale, residents living near fire-adapted public forests may recognize the benefits from policies for increased use of prescribed and natural fire to restore fire resiliency, but object to smoke and other byproducts that have deleterious effects on air quality [14]. Several prescribed fires have escaped control measures and, in some cases, resulted in catastrophic losses and subsequent tightening of fire management regulations [16–18]. Risk governance conflicts among large landowners, public land agencies, and communities will become more acute in the American West where urbanization and amenity production increasingly compete with traditional land uses. At the same time, individual wildfire events increasingly intersect parcels with landowners having diverse attitudes, fire management policies and risk governance systems, and changing current fire management policies is slowed by substantial “institutional momentum” [14, 15].

The interdependence of risk between different land owners and parcels within public land is recognized in newer US “all lands” wildfire policy (e.g. US Cohesive Wildfire Management Strategy [11]), and management initiatives increasingly emphasize collaborative, cross-boundary solutions to fire management [19, 20]. Here we define relevant boundaries as those delineating not only land ownership, but also the potential to manage forests and fuels by mechanical means (henceforth land tenures). However, mapping the scale of risk, quantifying risk transmission and measuring wildfire connectivity and interdependence across land tenures are largely unexplored domains. The study of risk transmission is widely discussed in the propagation of infectious disease in humans, plants, and animal populations [21] where, for instance, one organism transmits infectious disease to another. In the case of wildfire, the origins and extent of the disturbance are well defined spatially (i.e. mapped within a few meters), compared to other natural disturbances (e.g. earthquakes and hurricanes), and its propagation is affected by fuel loading on individual parcels [22]. A few studies have examined wildfire risk transmission from public lands to the wildland urban interface [23–26], but have not examined community specific wildfire transmission on large, mixed-tenure landscapes. For instance, how is wildfire risk shared and are there hotpots of risk transmission associated with specific land tenures and locations?

To understand the importance of risk transmission and help create assessment methods that can advance concepts for cross-boundary risk governance, we studied the exchange of wildfire exposure among large land tenures (federal managed, federal wilderness, private industrial forest, and state lands), and 56 adjacent communities on a 3.3 million ha fire prone area of the western US. We use the term exposure rather than risk since we limit our analyses to area burned rather than predicting expected loss [27]. Since most area burned by large wildfire events is under extreme burning conditions, we can assume that measures of burned area translate to high severity wildfire and loss of values on burned parcels. We used simulation modeling and network analysis to examine five specific questions: (1) to what extent do large tracts of federal, state, and private lands transmit wildfire exposure to each other; (2) do some land tenures transmit more wildfire exposure than they receive; (3) how many unique land tenures contribute to the wildfire exposure of typical western US communities; (4) what is the relative influence of human versus natural ignitions in the transmission of wildfire exposure; and (5) are there substantial differences in fire exposure among the different land tenures as described by fire intensity and likelihood?

Our study sets the stage for improving cross-boundary community, collaborative, and national forest planning [19, 20, 28, 29], with analyses that can help identify conflicts and opportunities to achieve federal wildland fire policy, including fire adapted communities, fire resilient landscapes, and wildfire response. Specifically we fill a gap in local, regional, and state-scale risk assessment methods [27, 30, 31] used for collaborative planning by providing a framework to quantify cross-boundary exposure and define the geographic scale of risk to communities. This information can be used to minimize scale mismatches between land tenures and risk planning at the community and landscape scales.

## Methods

### Study area

**Fig 1:**
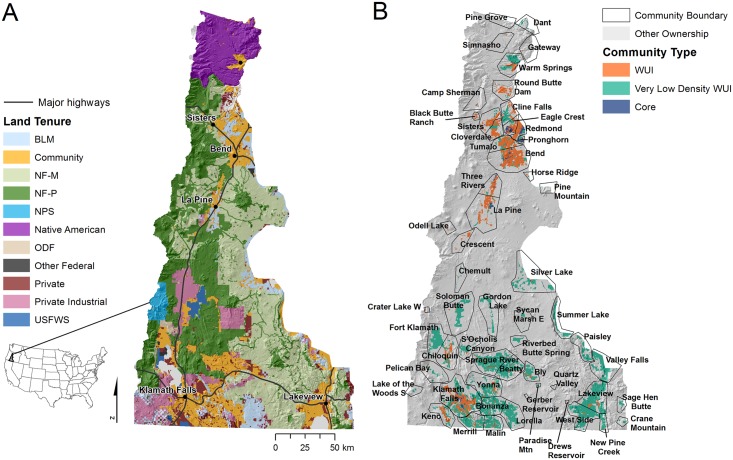
Study area maps of land tenures and communities. (A) Land tenures (defined by ownership and management practice), towns, and major highways. See Table 1 for land tenure descriptions. (B) Communities as defined by US Census data on core community area and surrounding SILVIS wildland urban interface (WUI) polygons. Community boundaries (light gray polygons) represent the combination of the core community area and associated WUI.

The study area was part of the “Forests, People, Fire” project [5] and spans 3.3 million ha in central Oregon, along the east slope of the Cascade range (Fig 1A). The physiographic gradients, forest vegetation, climate, and public management practices regarding wildfire management resemble the national forests throughout the western US, and are described in detail elsewhere [5]. The study area includes the towns of Redmond, Bend and Klamath Falls and many other small rural communities. Elevation ranges between 500 and 3240 m and the topographic-moisture gradient from west to east has a strong influence on the distribution of major ecological zones. Higher elevation regions to the west are dominated by mountain hemlock (*Tsuga mertensiana*) and fir and spruce subalpine forest, while mid elevation cooler slopes are characterized by moist mixed conifer patches of Douglas-fir (*Pseudotsuga menziesii*), grand fir (*Abies grandis*), white fir (*Abies concolor*) sometimes intermingled with lodgepole pine (*Pinus contorta*), and ponderosa pine (*Pinus ponderosa*) at lower elevations. High elevation plateaus on the east side are dominated by ponderosa pine mixed with semi-arid juniper (*Juniperus occidentalis*) woodlands and arid shrublands. About 30% of the study area is dry mixed conifer, while arid areas and juniper woodlands represent 19%. Stands of moist mixed conifer forests, ponderosa pine and lodgepole pine account for 9%, 14% and 5% of the study area, respectively. Alpine and high elevation non-forested areas and non-vegetated areas represent 10% and 13% of land use, respectively. The area is noted for extensive contiguous stands of low density ponderosa pine old growth that were maintained historically with periodic natural fire [32]. Many of these forests have developed dense understory conditions and ladder fuels largely due to policies that prioritize suppression of natural fires [28] and limit harvesting to preserve old forest habitat for wildlife and recreational amenities. Recent large fires include the B&B Complex in 2003 (36,733 ha), Pole Creek in 2012 (10,844 ha) and Sunny Side in 2013 (21,448 ha). The study area has also experienced rapid population growth and expansion of urban interface/intermix around several core areas including Bend, Redmond, Sisters, and Klamath Falls, contributing to a growing conflict between land management objectives of public forests and amenity interests of newer residents.

### Land tenure and community data

The major land ownerships in the study area are the federal government (66%, 28% protected), Confederated Tribes of Warm Springs (native, 8%), and private industrial (7%) (Fig 1A, Table 1). Private industrial lands are generally managed for wood products and are harvested on a regular rotation to maximize revenue. The Bureau of Land Management (BLM) and non-industrial private owners own roughly 6% and 4% of the area respectively. The latter are relatively small tracks of land ranging from 5 to 100 ha. Oregon state forested lands account for 1% of the study area. Federal land includes the Deschutes and Fremont-Winema National Forests, Crater Lake National Park (NPS), BLM and areas managed by the US Fish and Wildlife Service (USFWS). A small portion (1%) of the landscape includes minor federal owners such as the Department of Energy and Department of Defense. The ownership data were combined with federal land management data to create a land tenure dataset using data sources from the “Forest, People, Fire” study [5]. National forest lands were partitioned based on land and resource management plans (e.g. [33]) into protected areas (various conservation and biodiversity reserves) versus those available for mechanical fuel treatments. All other federal land with the exception of national park land was considered available for mechanical fuel treatment. The separation of managed versus non-managed was then incorporated into the land tenure classification.

**Table 1:** Major land tenures and transmitted wildfire estimated with wildfire simulation.

Land tenure	Description	Area (100 ha)	Total (%)	Total fire (ha yr^-1^)	Annual area burned (ha yr^-1^)					
					Human			Natural		
					NonTF	TF-IN	TF-OUT	NonTF	TF-IN	TF-OUT
BLM	Bureau of Land Management	2050	6	1377	233	406	410	283	456	465
Community	Core communities + wildland urban interface	4338	13	2580	496	905	826	352	828	698
NF-M	National forest, managed (e.g. general forest, timber production)	9350	29	4559	1139	679	764	1791	950	1094
NF-P	National forest, protected (e.g. biodiversity reserves, wilderness)	9280	28	3996	1056	709	777	1429	801	1085
NPS	National Park Service	390	1	72	4	9	2	37	23	24
Native	Native American (Confederated Tribes of Warm Springs)	2460	8	6706	3738	525	487	2000	443	351
ODF	Oregon Department of Forestry	180	1	66	5	36	21	4	21	16
OtherFED	Other federal land (e.g. Department of Energy)	290	1	423	59	138	124	53	172	93
Private	Non-industrial private	1450	4	829	70	337	304	50	371	223
Private Industrial	Corporate forests managed for wood products, harvested on regular rotation	2140	7	882	299	269	314	106	208	175
USFWS	US Fish and Wildlife Service	300	1	129	25	37	34	19	47	57

Total fire = nonTF + TF-IN. NonTF = the annual area of non-transmitted fire (self-burning). TF-IN = the annual area burned from incoming fires ignited on other land tenures. TF-OUT = the annual area burned on other land tenures by fires ignited locally. Additional descriptions of the calculations for transmitted fire are in the methods section.

In addition to the land tenures listed above, we created a community class based on core communities and surrounding sparsely populated wildland urban interface (WUI) areas. We defined core communities within the study area according to the US Census data on community boundaries [34]. Fifty-six communities were identified in these data representing 233,000 people and 118,000 structures. We then attached surrounding SILVIS wildland urban interface (WUI) [35] polygons outside the core community boundary based on distance to the closest community core. We removed SILVIS polygons that were: (1) classified as uninhabited, (2) classified as water, or (3) < 0.1 ha in size. The criteria for removing polygons conserved even the lowest density WUI areas adjacent to national forests. All other WUI polygons were assigned to a community and each WUI polygon was attributed with housing unit density (hereafter referred to as structures) and area (ha). The resulting community layer consisted of both the core area defined in the US Census and the adjacent WUI (Fig 1B).

### Wildfire simulation

Analyzing cross-boundary exposure from wildfires requires spatial information on ignition locations and fire perimeters. Broad geographic patterns of cross-boundary exposure have been previously described with historical fire databases for large areas (e.g. western US, Fig 8 in Ager et al. [36]). However, data on historical large fires, including perimeters, are only available at best for the past 20–30 years for much of US, and thus are insufficient to map and predict future patterns of potential wildfire transmission at the scale considered in this study. A solution is wildfire simulation, which is now a widely used practice for both tactical and strategic wildfire management in the US and elsewhere [37–40]. Monte Carlo simulations of many fires (>100,000) using historical weather and calibrated models can be used to generate sufficient fire samples to map fine scale transmission among parcels on >2 million ha with desktop computers. Most practitioners in the US use the minimum travel time (MTT) algorithm [41] that can replicate large fire perimeters and fire intensity patterns [37, 42–46]. We used a version of MTT encapsulated in FConstMTT, a command line version of FlamMap [41]. Fire simulations used a spatiotemporal fire prediction system using the methods of Preisler et al. [47] and described in detail elsewhere (S1 Appendix). The spatiotemporal ignition prediction model was built using 18 years of ignition data [48] to calculate: (1) the probability of a fire in a given pixel as a function of daily energy release component (ERC), location (X,Y coordinate) and day-of-year; and (2) the expected fire size for locations where the above-mentioned probability equals one as a function of daily ERC and location. We separated human caused ignitions from natural caused ignitions (S1 Appendix).

We executed the spatiotemporal ignition model to generate 3000 fire seasons for a total of 63,736 simulated fires, sufficient to saturate the landscape with wildfire and burn every pixel at least 10 times. The fire lists generated by the spatiotemporal ignition model used by FConstMTT specify ignition location, day-of-year, year, cause, ERC, wind speed, wind direction, fuel moisture, burn period and expected fire size of each fire simulated.

**Fig 2:**
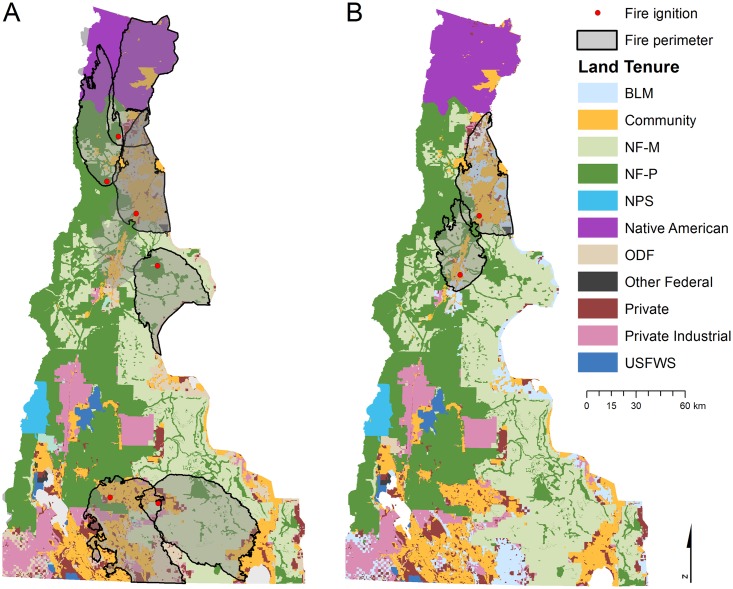
Simulated fire perimeters and ignition locations and associated land tenure map. Perimeters and ignition locations of (A) six of the largest fires based on area burned, and (B) two of the most destructive fires based on exposure to the most number of structures. Perimeters were extracted from over 63,736 simulated fires sampled from distributions modeled from historical ignition location and fire size. See Table 1 for land tenure descriptions.

Burning conditions associated with each ignition were sampled from historical days based on daily ERC. Fuel moisture for each fuel size class and fuel model were averaged over historical conditions (1987–2011) for each value of ERC, while wind speed and direction were based on daily ERC but restricted to gust observations in days of the historical record (1994–2011) where fires exceeded 500 ha in size. All weather data were obtained from the Lava Butte Remote Automated Weather Station (RAWS, [49]). We estimated the burn period (required by MTT) to achieve a specific fire size using a relationship between burn time and fire size built from controlled simulations (S1 Appendix). Simulated wildfire perimeters bore a close resemblance to historical fires within the study area [26]. Example large fire perimeters from the simulations show the potential impacts in terms of area and structures affected (Fig 2).

We obtained surface and canopy fuel for wildfire simulations from the national LANDFIRE dataset [50], as well as elevation, slope, aspect, fuel model [51], canopy cover, canopy base height, canopy height, and canopy bulk density [52]. LANDFIRE is a standardized fuel dataset available for the conterminous US and widely used for wildfire modeling and research in the US [50, 53].

FConstMTT simulations were performed at 90 m under the conditions specified in the firelists, and generated: (1) fire perimeters, (2) annual burn probabilities, and (3) conditional burn probabilities for 20 0.5 m flame length categories. The burn probability (BP) for a given intensity class is the ratio of the number of times a pixel burned to the total number of fires simulated and estimates the annual burn probability at each pixel. The conditional burn probabilities are output as a gridded point file and estimate the probability of a fire at a given intensity. A probability weighted average conditional flame length (CFL) was calculated from the conditional burn probabilities to measure the expected flame length given a pixel burns.

### Wildfire transmission between major land tenures

We calculated transmitted wildfire exposure using methods developed in our previous studies [54, 55]. Specifically, fire perimeter outputs and ignition locations were intersected with the land tenure and community map (Fig 1). We then cross-tabulated total area burned in each land class and community by ignition source to derive the amount of incoming (TF-IN), outgoing (TF-OUT), and non-transmitted fire (NonTF). The resulting data were then used to build transmission networks to analyze the connectivity among specific land tenures and into communities. Network construction and analysis were done in R [56] using the *igraph* package [57].

Networks are comprised of nodes and edges. In this study nodes corresponded to land tenures and communities while the edges represented directional transfer of fire (i.e. directed weighted network). Nodes represented multiple polygons for a given land designation; hence the edges measure the aggregate transmission of all the polygons of one land tenure to the polygons of other land tenures. Networks were analyzed at two ‘scales’: the scale of large land tenures and, the scale of individual communities. We separated transmission originating from human and natural ignitions within each network. A brief overview of network terminology used in this analysis can be found in Table 2.

**Table 2:** Organization of risk governance and wildfire transmission into networks.

Function	Network representation	Network scale	Risk governance implications
Spatial definition of landscape parcel in terms of land tenure	Node	Node	Defines landscape organization and the scale for measuring wildfire transmission and shared risk
Number of wildfire linkages among land tenures	Node degree	Node	Potential complexity for managing fire exposure transmitted to and from surrounding land tenures
Wildfire exchange between a pair of land tenures	Directed edge	Node	Magnitude of risk sharing among two land tenures
Number of incoming wildfire linkages for a land tenure	Node in-degree	Node	Number of surrounding land tenures that contribute to local exposure
Number of outgoing wildfire linkages for a land tenure	Node out-degree	Node	Number of surrounding land tenures that receive fire from the local land tenure
Overall wildfire connectivity among land tenures	Network density	Network	Measures the proportion of observed wildfire connections among land tenures to the total possible
The importance of a particular land tenure in the network	Centrality	Node/network	Priority land tenure for risk mitigation

Network wide statistics describe landscape-scale properties related to wildfire connectivity and sharing of wildfire exposure among land tenures. Communities are considered a specific land tenure category in the table for simplicity. Node statistics are descriptors of individual land tenure exposure to wildfire in terms of amount, number of sources, and overall connectivity to other land tenures. The combined analyses of node and network-wide statistics can facilitate community and landscape planning by revealing the scale and connectivity of wildfire disturbances.

We calculated whole network and node-specific measures pertaining to the frequency and strength of linkages. The node-degree measures the number of linkages for each node and indicates how central a node is within the network and is often interpreted as an indicator of connectivity and influence [58]. In the case of wildfire, node degree represents the number of landowners that contribute wildfire to a specific parcel, and the overall potential complexity of managing and resolving cross-boundary exposure issues for particular communities or parcels of land. Network density describes the number of nodes present in the network compared to the maximum possible, which represents the overall connectedness in the network. We also calculated the number of linkages transmitting fire into the land tenure (in-degree), and the number of linkages the land tenure was transmitting fire to other nodes (out-degree). Node degree is the total number of linkages for a node (land tenure), while in-degree and out-degree are the number of incoming or outgoing linkages. Networks were constructed and analyzed at two scales of aggregation. The coarser-scale grouping described transmission between larger landowners with communities aggregated into a single land tenure. The finer-scale grouping included the same large land tenures and individual communities.

### Housing exposure and community fireshed characterization

Community wildfire exposure occurred where simulated fires coincided with developed areas (i.e. the core community and the surrounding wildland urban interface). We intersected fire perimeters generated from the fire simulations with community polygons and then calculated the number of structures in each community potentially affected. The structure estimates were calculated as the product of the proportion of area of each community polygon burned and the structure count for that polygon. Structures were grouped by community (n = 56) allowing a single ignition point to create wildfire exposure in multiple communities.

We use firesheds to explore the spatial scale of community fire exposure within the study area. We define community firesheds as the areas that are likely to transmit wildfire to communities [36]. Using the simulation outputs described above we created a continuous smoothed surface of structure exposure for each community using the logistic kriging algorithm in the R *gstat* package in R 3.1.1 [56, 59]. Kriging is based on a semi-variogram that captures observed spatial dependence between points [60, 61]. Since the kriging algorithm is generalizable we used it to capture occurrence data as a binary logit function.

The kriging surfaces describe the predicted likelihood that an ignition occurring at a given point would expose structures to wildfire within that community (i.e. values varied between 0 and 1). To convert the kriging surface to a discrete boundary (i.e. the fireshed) we used a threshold of 0.1 meant to capture ignition locations where there was at least a nominal (10%) chance of exposure. To account for areas in the fireshed where ignitions were rare or absent we removed areas where the ignition point density was very low (i.e. less than 1.5e-4 km^-2^). Finally, community firesheds were intersected with the land tenure map to calculate the fireshed area by land tenure, and the proportion of the study area that transmits fire to the WUI. Our method for delineating firesheds, as compared to convex hulls used by others (e.g. [62, 63]), eliminates outlier ignitions that rarely threaten communities and contribute to larger fireshed sizes.

## Results

### Historical fire activity and ignition patterns

**Fig 3:**
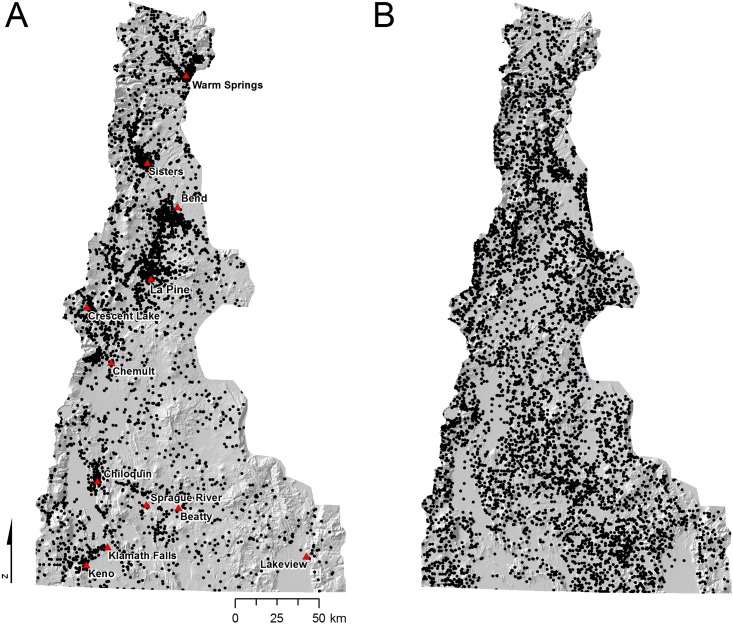
Spatial distribution of historical ignitions by fire cause. (A) Human and (B) natural caused ignitions in the study area based on historical ignition data between 1992 and 2009 [64], totaling 11,618 ignitions.

**Fig 4:**
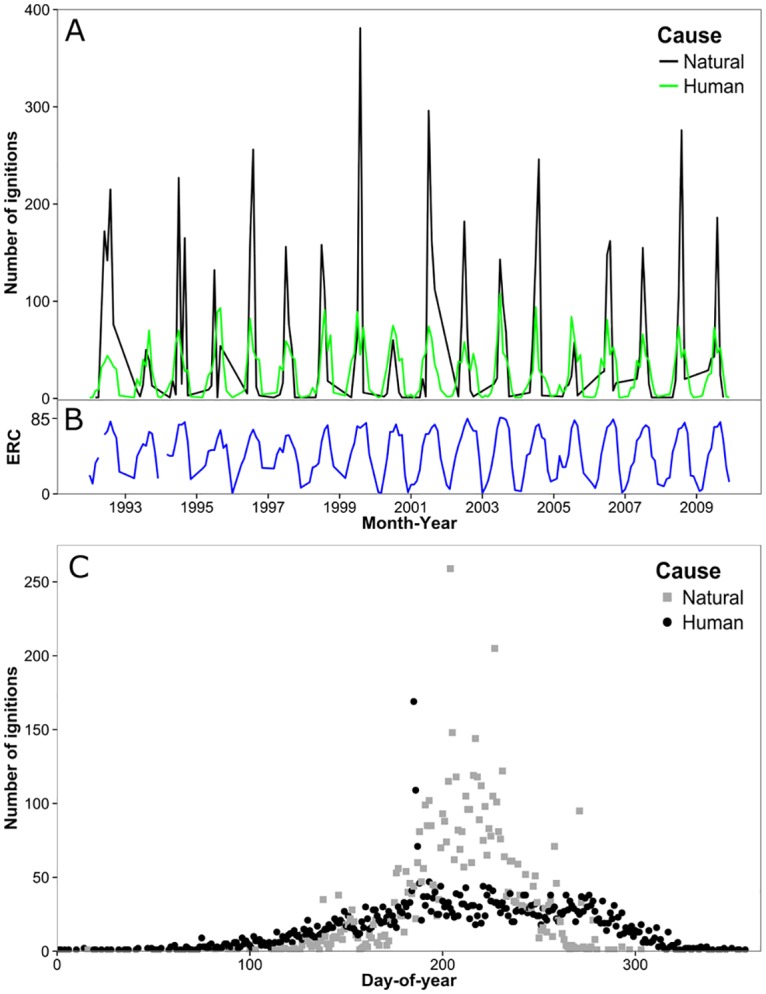
Temporal and seasonal distribution of historical ignitions in the study area. (A) Monthly distribution of total number of human and natural caused ignitions based on historical ignition data between 1992 and 2009 [64], and (B) associated monthly maximum energy release component (ERC) for the same time period based on 25 remote automated weather stations [49]. (C) Total number of ignitions by day-of-year and fire cause during the same time period.

Analysis of historical fire data provided insights into the seasonality and spatial distribution of wildfires and the relative frequencies of human versus natural ignitions and resulting burned area. The results confirmed that both human and natural ignitions contributed substantially to area burned, and that each ignition source required a different spatiotemporal model for the wildfire simulations. About 16,061 ha of wildfire burned within the study area each year annually based on 11,618 ignitions (645 ignitions yr^-1^) occurring between January 1992 and December 2009 (Fig 3). Lightning resulted in roughly the same burned area as human caused fires (7,877 ha versus 7,184 ha yr^-1^) although with more ignitions (6,379 or 354 ignitions yr^-1^ versus 5,239, or 291 ignitions yr^-1^). Lightning caused ignitions (hereafter natural) had a slightly smaller average fire size (22 ha) compared to human (26 ha). Ignition locations from humans and lightning differed substantially (Fig 3). Human ignitions were clustered near towns and cities within the study area, especially Warm Springs, La Pine and Bend. Natural ignitions were frequent in the south-central and southeast portion of the study area where human ignitions were relatively sparse. Temporal variation in ignitions was also pronounced (Fig 4). Natural ignitions peaked in the middle of the summer months (Fig 4C) whereas human ignitions were relatively more frequent in the spring and fall.

### Simulated fire activity

**Fig 5:**
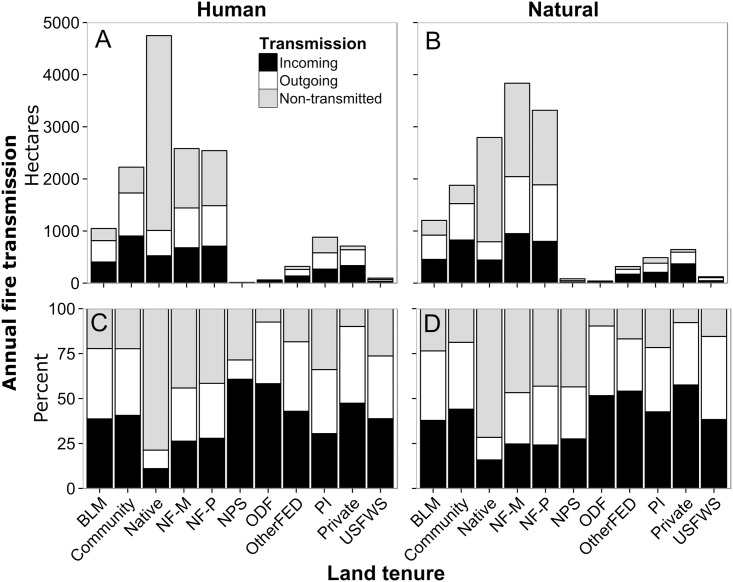
Wildfire area burned by land tenure classified by transmission type and fire cause. Total annual fire transmission from human (A, C) or natural (B, D) caused ignitions. Data shown both as the average annual area burned (A,B), and the percentage of total fire transmitted (C, D). See Table 1 for land tenure descriptions.

We summarized simulated fire activity to examine broad trends in relation to historical fires. Simulated fires burned 21,650 ha yr^-1^: 11,200 ha yr^-1^ were the result of human caused ignitions; 10,460 ha yr^-1^ burned due to naturally caused ignitions. Fire was most common on national forests (8,554 ha) and native lands (6,710 ha), which together accounted for 72% of all land burned. More than one-third of the area burned (8,402 ha, 39%) resulted from wildfire events that spanned at least one boundary. Human caused fires burned comparatively more area of native, community, private and private industrial land tenures (Fig 5A; Table 1). This difference was most pronounced for native lands and least for the private land tenures. Fires from natural ignitions were responsible for a comparatively larger area burned on national forest and BLM lands.

**Fig 6:**
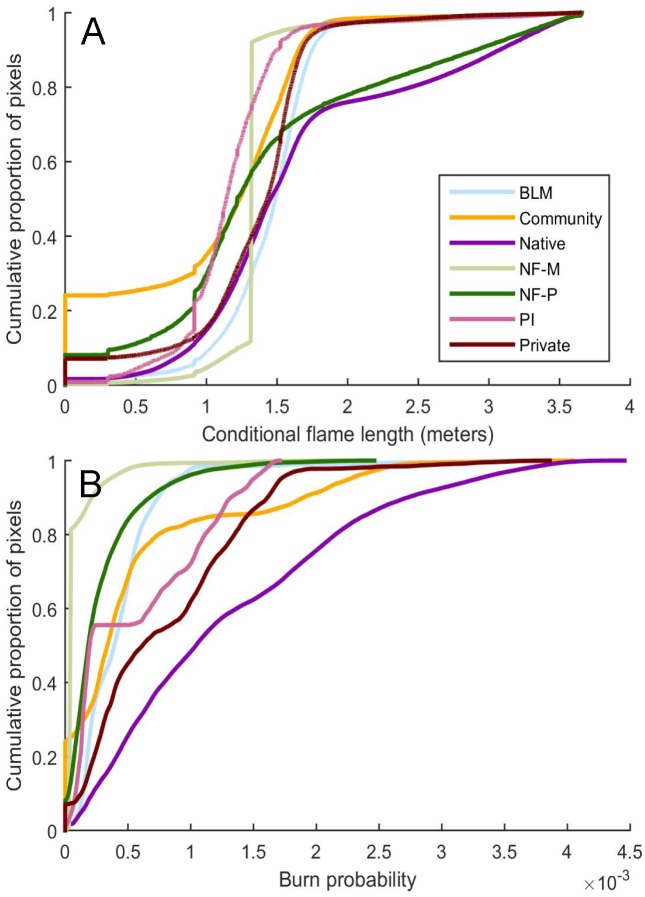
Variation in fire hazard for the seven largest land tenures. Cumulative proportion of pixels in the study area by (A) conditional flame length, and (B) burn probability for the seven largest land tenures in the study area. See Table 1 for land tenure descriptions.

We characterized wildfire hazard for each land tenure to understand if different management practices (conservation versus active forest management) have resulted in distinct expected fire behaviors. Hazard was defined as the potential flame length given a fire as described in the methods. The largest land tenures presented similar hazard profiles for the majority of their area, i.e. roughly 60% of each land tenure was expected to burn with a flame length <1.5 m (Fig 6A). Fire hazard for the remaining proportion varied by land tenure, with higher hazard on native and protected national forest lands (NF-P), and lowest hazard on private industrial (PI) and managed national forest (NF-M) land tenures. By contrast, burn probability profiles (Fig 6B) were distinctly different across land tenures reflecting inherent spread rate, potential fire size, and ignition probability. The highest burn probability profiles were associated with native and both non-industrial and industrial private lands. Low burn probability profiles were observed for national forest managed lands, national forest protected lands, and BLM.

### Fire transmission between major land tenures

The relative and absolute amount of area burned by each wildfire was partitioned into incoming, outgoing, and non-transmitted fire to analyze transmission characteristics for each land tenure. Note that a single fire event can contribute to both non-transmitted and transmitted fire. Among the land tenures, total area burned by incoming fires averaged 57% and varied from a low of 14% to a high of 86% (Fig 5C and 5D). For communities, the average amount of area burned by incoming fires was 67%. On a percentage basis, the relative amounts of different transmission types (in, out, non-transmitted) changed mostly because of variation within land tenures versus incoming fire from other sources; outgoing fire was relatively constant (about 25%) with some exceptions (e.g. Native, NPS). We did not find large differences in transmission patterns by ignition type (human versus natural, Fig 5A and 5C versus 5B and 5D), although on a percentage basis national park lands (NPS) had more area burned by incoming fire from human ignitions, and private industrial had more incoming for natural ignitions (Fig 5C and 5D; Table 1).

**Fig 7:**
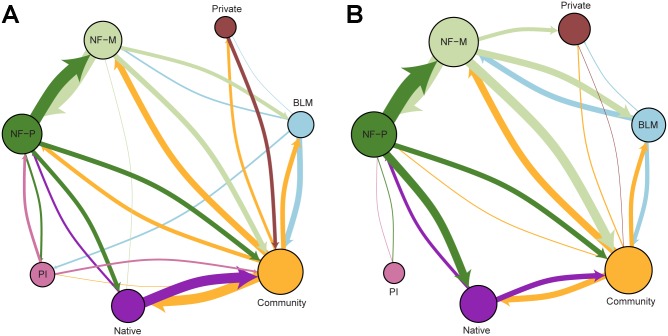
Wildfire transmission networks for the seven largest land tenures. Networks show wildfire transmission between the seven largest land tenures in the study area, separated by human (A) and natural (B) ignitions. Network edges represent wildfire transmitted from one land tenure to another, as shown by the arrow and colored by its source. The size of each node corresponds to the amount of fire transmitted from that node. Only edges greater than 20 ha yr^-1^ are shown, representing 75% of the all transmitted fires. See Table 1 for land tenure descriptions.

The fire transmission network shows that fire was transmitted between all 11 land tenures via 129 network edges (i.e. directed edges), corresponding to a network density of 0.977 (meaning that 97% of all possible linkages between land tenures were present). Connectivity of individual nodes in the land tenure network, as measured by node degree, varied from a low of 12 to a high of 24 (for the full network). Land tenures with lower node degree receive and transmit fire to a lesser extent than land tenures with higher node degree. Fig 7 shows fire transmission between the seven largest land tenure classes by ignition type for transmission pathways greater than 20 ha yr^-1^ representing 75% of all transmitted fires.

National forest was the greatest source of transmitted fire within the study area, although most fire was exchanged within the national forest between managed (NF-M) and protected areas (NF-P) (Fig 7, green nodes). While fires originating on national forest lands moved into community lands (50% more from naturally caused ignitions), fires that started on community lands also moved into the national forests (5% more from human caused ignitions). The largest exchange of fire with community lands was with native lands, particularly notable concerning human caused ignitions, followed by managed national forest (NF-M). The maximum outgoing (directed edge) value was observed for fires that originated from protected national forest land (NF-P) and burned 818 ha of managed national forest.

### Fire transmitted into communities

**Fig 8:**
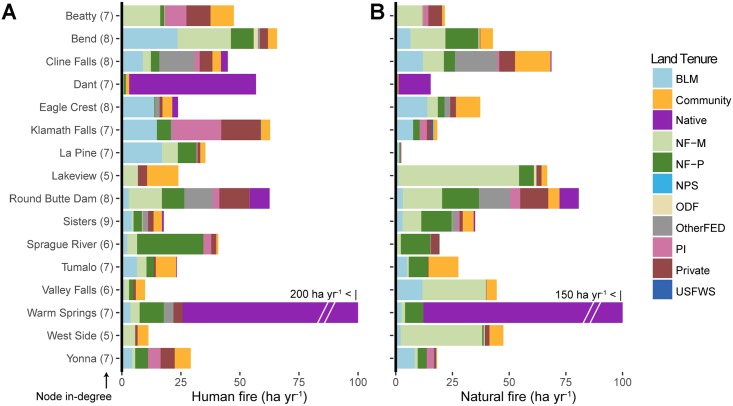
Community exposure to external wildfire by fire cause. Total annual area of fire transmitted to the 16 communities within the study area that received the most fire, separated by (A) human and (B) natural ignitions as estimated from simulation modeling. Wildfire received from adjacent lands is colored by large land tenure. Numbers following community names indicate node in-degree (number of neighbors transmitting fire). See Table 1 for land tenure descriptions.

Quantifying wildfire transmission into communities revealed the amount and relative contributions of human versus natural ignitions and the major contributing land tenures. Twenty-one percent of the total wildfire transmitted among land tenures burned into communities. These incoming fires accounted for 67% of the total community exposure as measured by average area burned. The total area burned in communities was predicted to be 1,400 ha yr^-1^ for human and 1,179 ha yr^-1^ for natural ignitions. The amount of fire received by each community varied (Fig 8, S1 Table) from a low of 0.1 ha to 136 ha yr^-1^. The number of structures exposed to fire also varied by community. For natural ignitions, the annual structures predicted to be exposed to fire was 205 structures yr^-1^ and ranged from 83 structures yr^-1^ for Bend to nearly 0 for Fort Klamath. For human ignitions, the annual number of structures predicted to be exposed to fire was 301 and ranged from 114 structures yr^-1^ for Bend to 0 for Fort Klamath and Crater Lake.

Transmission of incoming fire to communities varied in terms of amount and relative contribution of the sources (Fig 8). There were instances where the communities received the majority of fire from each of the major land tenures including managed national forest lands (e.g. Lakeview), protected national forest (e.g. Sprague River, Sisters), private industrial (e.g. Klamath Falls), and native (e.g. Warm Springs, Dant). The amount of area burned by ignition type (i.e. human versus natural) differed for most communities (e.g. Lakeview, La Pine) but less so for others (e.g. Bend, Warm Springs).

**Fig 9:**
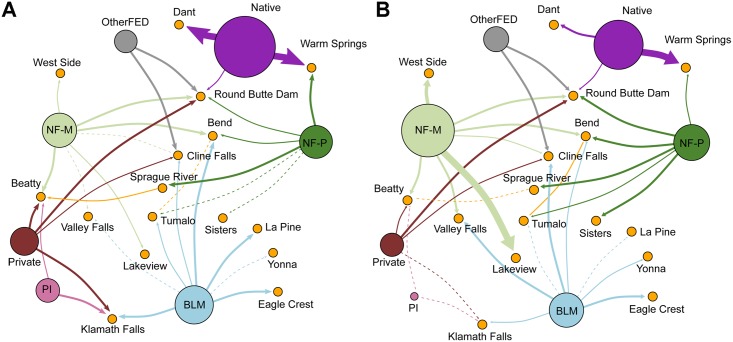
Wildfire transmission networks from major land tenures to communities. Wildfire exposure to the 16 communities that received the most fire from adjacent land tenures in the study area, divided between (A) human, and (B) natural ignitions. Network edges represent wildfire transmitted from one land tenure to a community, as shown by the arrow and colored by its source. Edge thickness corresponds to the magnitude of transmission. Solid lines show wildfire transmission >10 ha yr^-1^ calculated by combining the ignition sources. Dashed lines represent edges with values <10 ha yr^-1^ and >1 ha yr^-1^ when separated by ignition source. Nodes of major land tenures are sized by amount of fire transmitted; community node size is fixed. Note that transmission between major land tenures is not shown. The network diagram corresponds to information shown in Fig 8. See Table 1 for land tenure descriptions.

**Fig 10:**
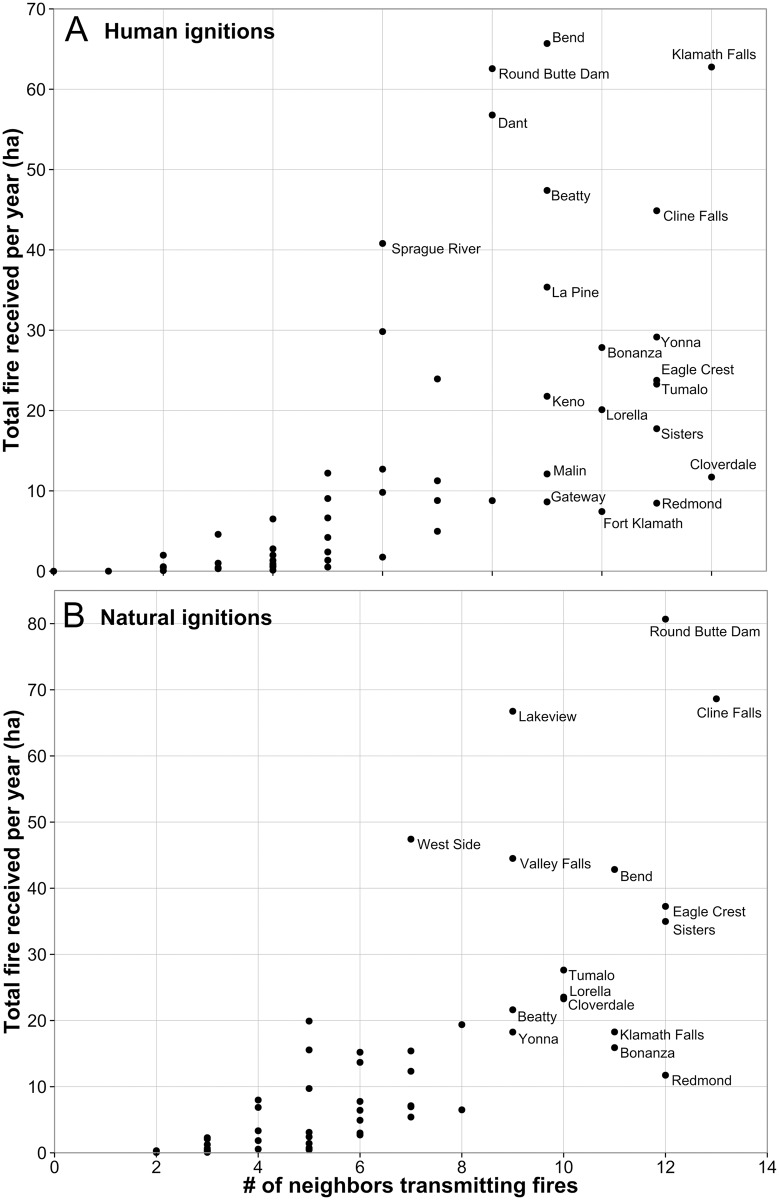
Relationship between community wildfire exposure and land tenure diversity. Number of land tenures transmitting fire (i.e. node in-degree) and total fire received (incoming fire) per year for the 56 communities in the study area for (A) human and (B) natural ignitions. Note that the outlier Warm Springs is not shown.

The community transmission network contained 66 nodes and 862 non-zero linkages; 20% of all possible linkages between land tenures and individual communities were present (network density = 0.2). When the network is filtered for substantial wildfire transmission (edges >10 ha yr^-1^), connectivity of individual nodes in the land tenure network varied from a low of 2 to a high of 15 (Fig 9). For instance, Crater Lake West and Odell Lake had only two connections, while Klamath Falls and Redmond had 15. Communities receiving more fire had higher node degree (Fig 10). In general, the node degree of communities was higher than for the large land tenures.

**Fig 11:**
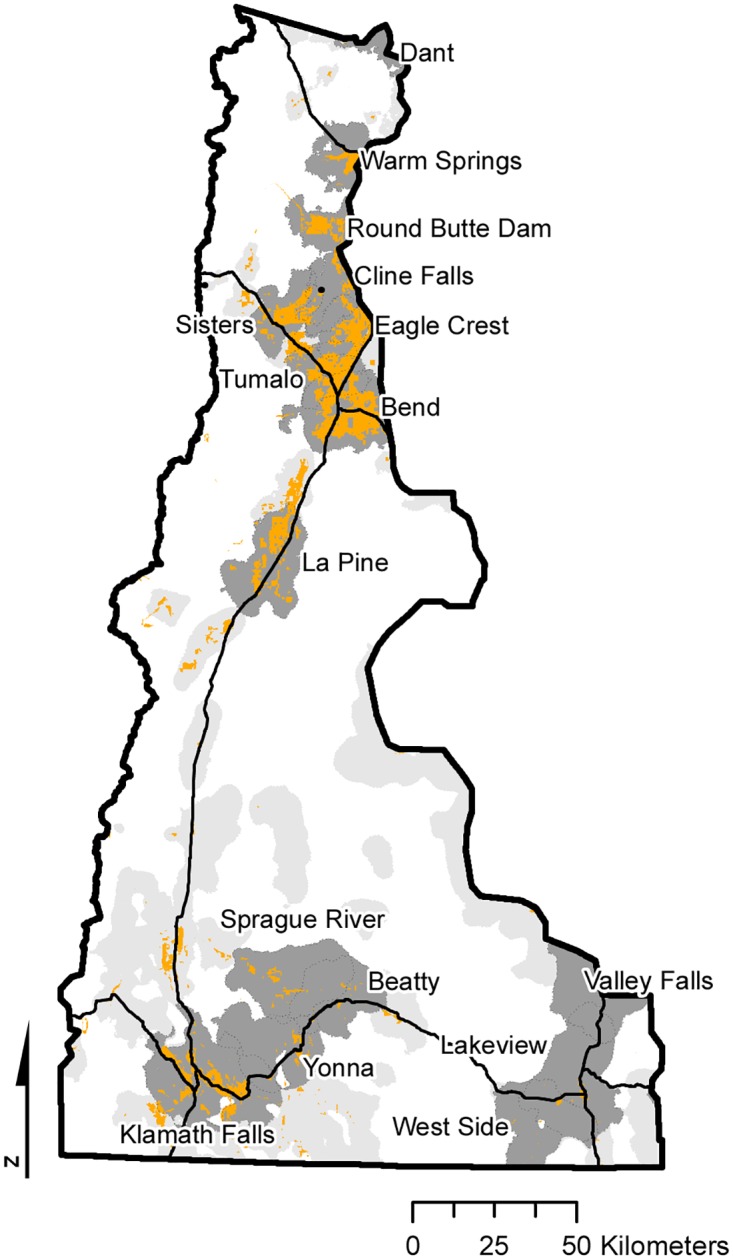
Community fireshed map. Fireshed map showing the area predicted to transmit fire into communities based on structure exposure. Orange = developed areas; dark gray = individual firesheds for the 16 communities that received the most fire; light gray = firesheds for the remaining 40 communities.

**Fig 12:**
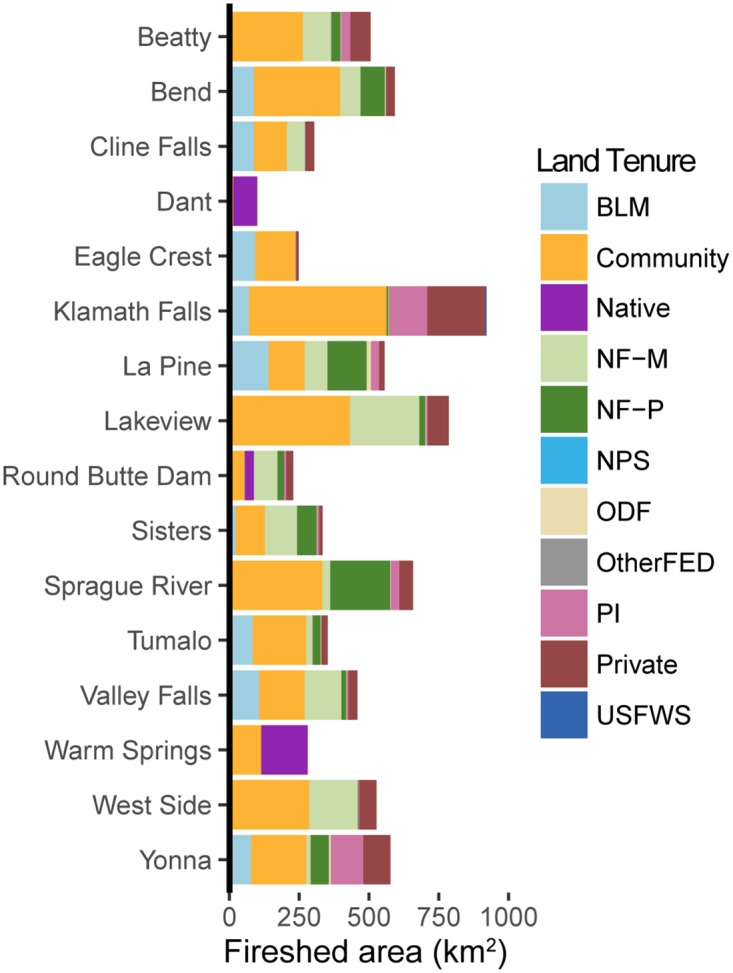
Community fireshed composition. Community firesheds represent the land tenure composition around each community most likely to contribute to fire exposure. See Table 1 for land tenure descriptions.

The analysis of community firesheds allowed us to map and characterize land tenures that were predicted to contribute cross-boundary wildfire exposure to communities. We found that these areas constituted 1.34 million ha, or 40% percent of the study area (Fig 11, S1 Table). While most community firesheds represented distinct geographies, 19% of the combined fireshed area corresponded to more than one community, thus the potential to inform collaboration among communities for wildfire protection planning was high. About 80% of the fireshed area was in fire adapted forests as defined by fire regime data [65]. The combined fireshed was 54 times the size of communities alone and 5.5 times the size of both the community and its surrounding urban interface. More than 40% (600,000 ha) of the community fireshed area was on federal lands, two thirds of which could be managed using mechanical treatments under current land resource plans while the remainder was located in amenity reserves, biodiversity areas, and national parks (NF-P and NPS, Fig 12).

**Fig 13:**
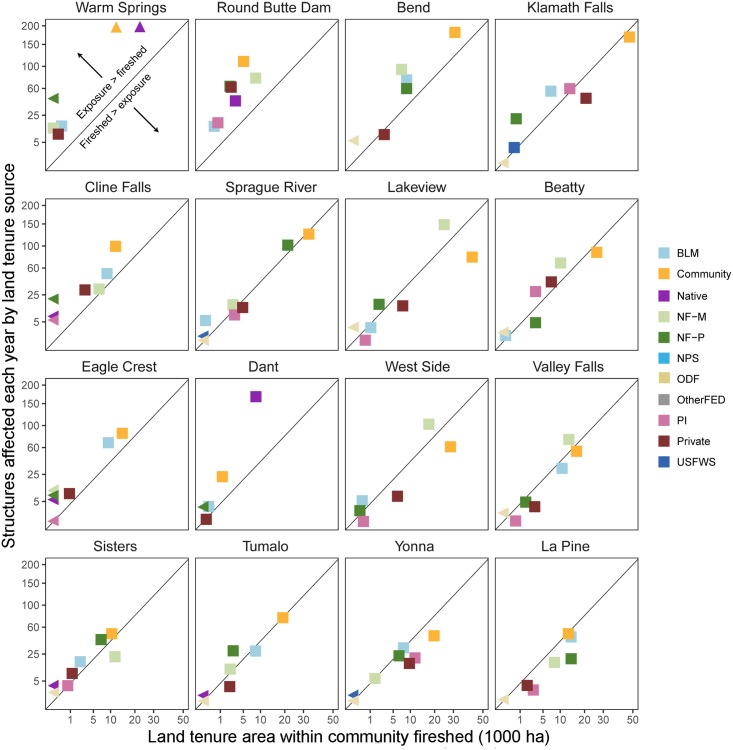
Comparison of community wildfire exposure and fireshed composition by land tenure. Each panel represents one of the 16 communities with the highest level of exposure. Diagonal line indicates an exposure of 2 structures per 1000 ha of fireshed area. Land tenures above the line have higher than this value, indicating high efficiency at transmitting fires to structures. Land tenures below the line indicate lower transmission per area in the fireshed. See Table 1 for land tenure descriptions.

We compared fireshed area versus structures exposed to identify firesheds that were efficient at transmitting fire to communities on an area basis. For the top 16 communities most exposed to fire (Fig 13) exposure tended to match fireshed composition (the more area burned, the more structures exposed), although the exact relationship varied by community (S1 Table). Wildfire exposure in Warm Springs, for instance, exceeded what would be expected from nearby lands alone, whereas West Side and Valley Falls were much less exposed than their surrounding fireshed would imply. Community exposure from a number of land tenures tended to fall outside the fireshed, namely protected national forest (NF-P) and BLM lands. These instances represent exposure from land tenures capable of producing particularly large cross-boundary fire events.

## Discussion

This study was motivated by newer US federal wildfire management policy that emphasizes cross-boundary “all lands” management of wildfire as a means to build fire resilient landscapes and fire adapted communities, promote the safe, effective use of wildfire [11], and improve wildfire suppression response. Similar cross-boundary risk management concepts are being advanced for fire policy in other fire prone countries as well [25]. Our methods advance these concepts into practical application by offering quantitative methods to assess cross-boundary wildfire exposure on landscapes fragmented by land tenures, wildland fuels, and community boundaries. Current risk assessment protocols [30, 31] do not identify or map cross-tenure and tenure-community fire transmission that underlie the “all lands” planning objectives [30]. More importantly, mapping fire risk [66] alone is insufficient to inform mitigation strategies [67] that must balance goals for improving fire resiliency in fire dependent forests versus protecting assets from fire. Our analysis provides key metrics for use in cross-boundary planning at multiple scales [19, 20, 28, 29], and illustrates where wildfire connectivity among land tenures needs to be addressed to achieve long range goals of federal wildland fire policy. Moreover, our spatial characterization of risk (i.e. risk transmission, defining scales, and mapping firesheds) can help reduce scale mismatches between local planning and potential wildfire events [68, 69]. At broader regional scales, mapping the scale of risk and cross-boundary exposure can help reduce fragmentation in existing risk governance systems described by Steelman [8]. Widely available fire simulation models [37] and input data for the conterminous US [52] and elsewhere [70, 71] make these methods and simulation outputs transferable to other areas (e.g. Ager et al. [23]).

We addressed five specific questions regarding risk transmission and fire behavior in our study area concerning the amount, composition (i.e. incoming versus outgoing fire) and scale of cross-boundary wildfire events from both human and natural ignitions. Simulation outputs indicated that over half of the annual area burned within each land tenure started in some other land tenure, i.e. cross-boundary fire. Of the total area burned in private and private industrial parcels, 69% of the burned area was from ignitions that started in other land tenures, and 67% of wildfire that burned into communities came from fires ignited in surrounding land tenures. There were only minor differences among land tenures in the relative percentage of incoming versus outgoing fire with exceptions noted for incoming fire for human ignitions within the Crater Lake National Park (NPS, Fig 5) and state of Oregon land (ODF). The broad differences in relative amounts of transmitted versus non-transmitted fire among the land tenures were due to a host of factors, including parcel geometry, relative parcel sizes, and differences among the parcels in terms of ignition probability and wildfire spread rates [26]. Analyzing the relative influence of these various factors on wildfire transmission is a subject of our future work.

We addressed the functional and spatial scale of risk to communities by measuring the number of contributing land tenures (node degree) and the area that exposes communities to wildfire (i.e. fireshed). Our results showed that each community has a unique fire signature in terms of the potential contributors to future fire events [72]. On average communities received fire from about five land tenures, with a low of 2 and high of 13. We found that the contribution from federal land tenures varied substantially among the communities, and 88% of wildfires reaching communities originated from areas that can be managed with mechanical fuels treatments, i.e. outside of wilderness and other protected areas. We also found that communities with a higher node degree (number of adjacent land tenures) had greater overall exposure. While the underlying reasons for this finding are unclear, it suggests that the complexity of planning as measured by the number of contributing land tenures is correlated with the magnitude of wildfire exposure to communities. Network connections between large landscapes and communities identify high risk landscapes to target for risk reduction investments, including fuel break systems [25, 73], creating fire resilient landscapes with restoration programs [28], and building suppression capacity in coordination with fuel treatments. Communities with higher complexity in terms of incoming fire will require more cohesive risk governance systems to facilitate coordination and collaboration among organizations that represent the various land tenures [20]. Empirical studies have shown that jurisdictional boundaries strongly influence the geographic scope of community wildfire planning [74–77], rather than boundaries defining the scale of risk. As noted above, current risk assessment frameworks do not quantify or consider land tenure composition, the magnitude of incoming fire to communities, or the exchange of fire among different land tenures (e.g. [30]).

The area in community firesheds (i.e. wildlands that transmit fire to communities) covered more than 40% of the study area—five times that of the total developed area, and 50 times larger than the core communities. Our fireshed delineations defined and mapped the scale of risk to communities, and provided a biophysical determination of risk exposure that is not considered in current community wildfire protection planning (CWPP) guidelines [78] where boundaries are based on ownership and administrative borders [76] rather than the spatial and functional scale of wildfire risk. These resulting scale mismatches (e.g. [9, 10, 79]) between CWPP boundaries and the scale of risk can contribute to ineffective outcomes [69]. In general, communities with high wildfire connectivity and large firesheds have a higher potential for scale mismatches in community planning stemming from poor perception of risk transmission [69, 80, 81]. Scale mismatches manifest in a lack of coordination between local communities and surrounding landowners where governance institutions fail to recognize transmitted risk from neighboring land ownerships. Although US CWPP [74, 76] mandates collaborative planning, guidelines for the structure and composition of the collaborators are vague in terms of defining actors and their relative responsibilities to mitigate risk [82]. For instance, reducing scale mismatches in CWPP can be facilitated by bridging linkages across organizations within collaborative planning groups [74, 75, 83] as identified in analyses of risk transmission. Social network analysis [84, 85] can be used to to identfy the need to improve specific bridges among agencies and landowner organizations that are not active in the planning processes, and improve social capacity and competence to effect risk reduction activities within communities [69, 86].

Additional characterization of community exposure will contribute to the development of fire transmission archetypes to organize communities based on amount of exposure, fire intensity, fire likelihood, contributing land tenures and fuel types. Similar approaches were used in fire prone regions of Europe [87]. The archetype concept was also found useful for organizing communities based on socioeconomic factors [88, 89]. Biophysical and social information can be combined to help prioritize and design restoration and fuel management programs to meet policy goals to create fire resilient landscapes and fire adapted communities. We expect archetypes will be useful to classify communities based on land ownership patterns, capacity to manage fuels, and public versus private sources of risk. For instance, one archetype might include the “checkerboard” ownership in the West due to railroad land grants that deeded alternating sections of land 10 km to 64 km wide along rail corridors. The resulting land pattern sets the stage for cross-boundary risk transmission.

Differences in wildfire exposure among each land tenure as determined by simulation outputs suggested higher potential flame length and lower burn probability on national forest lands (both protected and managed). Lower burn probability could be due to either spread rate or ignition probability or both. The highest burn probability profiles were associated with native and both non-industrial and industrial private lands. Some of the observed differences in potential fire behavior among the land tenures are due to the respective fuels, where national forest lands are more heavily forested with slower spreading fuels that burn at high intensity compared to lower elevation areas with a higher composition of grass and shrub fuels with high spread rates and lower fire intensity.

Our analyses of human caused versus natural ignitions revealed the relative influence of human versus natural ignitions on the origin of wildfires and resulting transmitted exposure. We found that historical lightning fires were slightly smaller than human caused fires (22 versus 26 ha) and that the two ignition types were responsible for about the same area burned both historically and predicted by simulation. These findings are in contrast to other studies in the region that reported that lightning fires were substantially larger and accounted for the bulk of the area burned [90]. It is important to note that our ignition prediction system predicted fires >10 ha which might have a slight effect on our estimate of the average fire size, but not a significant effect on the estimate of area burned. We observed distinct spatiotemporal patterns of human versus natural ignitions (Figs 3 and 4), as documented in numerous other studies [90, 91]. The most significant finding of this analysis is that human ignitions play an important role in the exposure of many of the communities, and that the relative importance of natural versus human ignitions as a source for incoming fire varies substantially among the communities (Table 1, Fig 5). Although human ignitions are the source of fires in other areas with high population density, (e.g. California, [91]), the finding is not typical for rural areas in the western US. Human ignition sources that generate cross-boundary wildfires can be mitigated with prevention programs [92] based on assessments and characterization of causal human behaviors (e.g., arson, field burning, recreation).

The application of network concepts served several purposes in the study. The framework and analyses helped underscore risk as a process rather than a static condition (e.g. map). Network diagrams can help researchers and planning organizations understand broad patterns of landscape fire connectivity that can facilitate discussions among stakeholders about risk governance. Network analysis is widely applied in conservation biology to measure landscape connectivity [93–97] and identify scale mismatches in planning [98]. In fire suppression activities, communication networks are critical to understanding incident response, and network analysis has shown where missing communication links could lead to cascading failures in suppression activities [68]. Network statistics (Table 2) have specific interpretation for fire exposure both at the node and network levels and these can each inform different scales of fire management planning. For instance, network level statistics are relevant for landscape-scale analyses of risk, exposure, and prioritization of fire protection and restoration activities. Node level statistics inform individual landowners and communities about the diversity and magnitude of transmission problems. Ultimately, by combining social and fire networks [6] the longer-term problem of wildfire risk can be analyzed as a socioecological system to identify the extent to which social and ecosystem resilience are compatible at the community scale [1, 67]. For instance, social network theory suggests denser networks engender better risk perception [1, 5], and thus dense social networks are needed where fire transmission networks are dense. Research is needed to understand how social networks reorganize both in anticipation of and in response to large wildfire events, and whether public policy can actually facilitate this process to accelerate adaptation to wildfires under a changing climate [99].

Finally, incorporating wildfire network analyses at the federal, state, and community scales can help eliminate functional and scale mismatches in wildfire risk management that reduce the effectiveness of existing risk governance. For instance, forest practices in the private sector are controlled by state level regulations, while the bulk of fire suppression is the responsibility of federal agencies. Community protection is governed by community planning guidelines that do not require cross-boundary assessments [100]. The challenge of designing an adaptive governance system for natural disaster planning, including wildfire, is matching governance regimes with disturbance processes at the scale at which they operate [74, 101]. Temporal, spatial, and functional scale mismatches between governance regimes and wildfire disturbances contribute to the pathology of wildfire risk management [1]. Heterogeneity in spatial patterns of fire transmission between land tenures illustrated in this study, combined with fragmentation in existing wildfire risk governance structures [8] can help perpetuate scale mismatches that ultimately reduce the effectiveness of mitigation planning. Scale mismatches are highly relevant to US wildfire management, and as discussed by Abrams et al. [74] and Ager et al. [69], result from complex interactions between governance and wildfire events. For instance, the short-term success of fire suppression policy leads directly to increased fire risk in the future and dependency on increased suppression expenditures [4, 102]. Another scale mismatch results from local land use policy that permits residential development in fire prone areas at the expense of national policies for wildfire suppression activities [103]. Yet another example comes from national forest planning were forests are fragmented into large numbers of land management designations (e.g. protected reserves, recreation sites, cultural areas) that are orders of magnitude smaller than large fire events. Long-term solutions to managing wildfire on mixed tenure, fire prone landscapes will require bridging the scales of various planning and governance systems with the increasing scale of large wildfire events.

## Supporting information

S1 Appendix Spatiotemporal fire prediction system. Description of statistical model used to predict fire occurrence, size and location, as well as weather conditions, that were used to parameterize a wildfire prediction system written in R outputting a fire list text file containing predicted fire weather, burn probability, burn period, fire cause and fuel moisture conditions for each day a fire occurs in each simulation year.
(PDF)

S1 Table Community fireshed area. Fireshed area showing the area predicted to transmit fire into communities based on structure exposure with associated wildfire and structure exposure values.
(PDF)
