# Supplementary material for: Network analysis of wildfire transmission and implications for risk governance
Source: PLoS One. 2017 Mar 3;12(3):e0172867. doi: 10.1371/journal.pone.0172867 (PMC5336224; doi:10.1371/journal.pone.0172867)
Supplement: S1 Appendix — Description of statistical model used to predict fire occurrence, size and location, as well as weather conditions, that were used to parameterize a wildfire prediction system written in R outputting a fire list text file containing predicted fire weather, burn probability, burn period, fire cause and fuel moisture conditions for each day a fire occurs in each simulation year. (PDF) [file pone.0172867.s001.pdf]

Appendix S1: Spatiotemporal fire prediction system

The spatiotemporal wildfire ignition prediction model was built using historical ignition and Energy Release Component (ERC) data to predict the probability and location of a fire and the expected fire size for both human caused and natural caused ignitions (Fig 1).

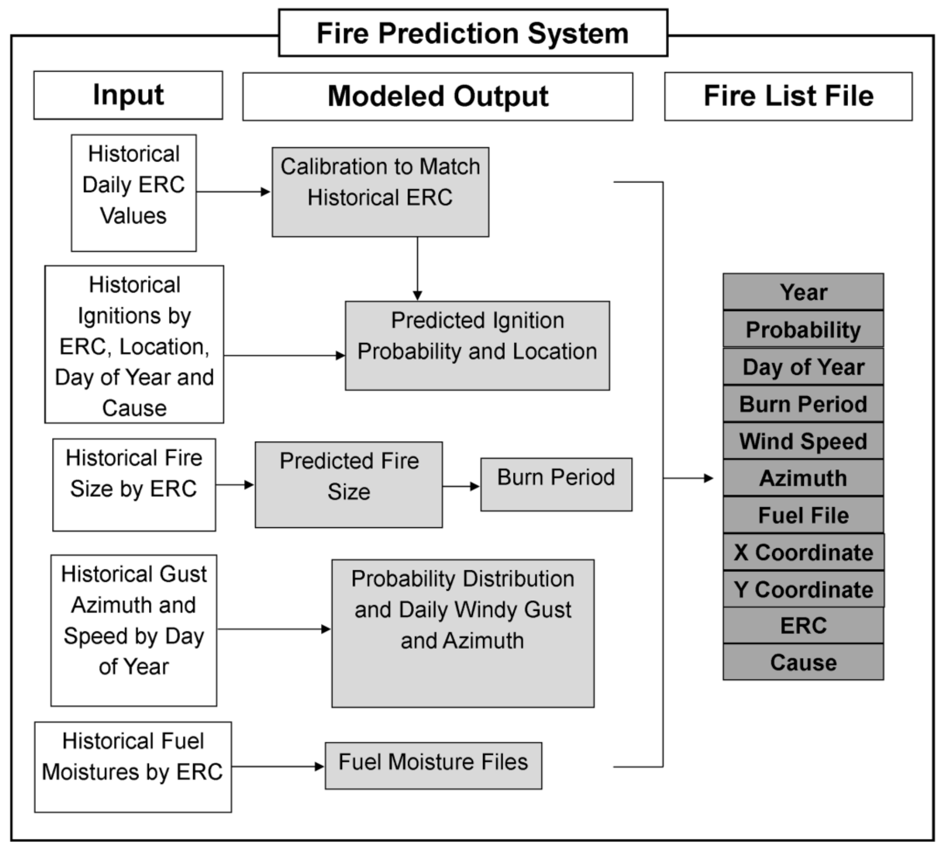

Fig 1. Diagram showing the major components of the spatiotemporal fire prediction system.
Flow chart shows the components of the fire prediction system including input variables and resulting fields in the fire list file that are read into FConstMTT.

Statistical model for predicting wildfires

We used empirically-derived relationships between energy release component (ERC) and historical fires to predict daily fire occurrence and fire size [1, 2]. The historical reference period for predicting fire occurrence was 1992-2009. Historical ignition data and cause (human or natural) came from the spatial wildfire database of the US [3]. There were 11,618 ignitions in the study area between 1992 and 2009. Daily ERC data were downloaded from the RAWS USA Climate Archive for 25 remote stations in the study area from 1961-2011 depending on the station [4]. Variability among the stations was relatively low and hence ERC values were

averaged by day of ignition. We developed independent models for fire occurrence versus fire size as described individually below.

Predicting fire occurrence

We estimated separate probability models for lightning (hereafter natural) and human caused fires, with spatial location, day-of-year and ERC as explanatory variables. In other words, the probability models are spatially (km^2) and temporally (day-of-year) explicit. We followed previous statistical modeling of wildfires and fire danger rating systems used in the US [5] that used ERC as a predictor [1].   Both models include a spatial term (to account for different spatial patterns of natural and human caused ignitions), a seasonal term (seasonal patterns for natural and human caused fires are different), and a term for ERC effect. Because we observed outliers (large number of ignitions) in some years due to large lighting events, and because none of the explanatory variables used in the model account for these lighting episodes, we included a random year effect in the model to simulate episodic lightning ignitions [8]. We used logistic regression with non-parametric smooth spline functions to estimate the terms in the model.

We used the Mixed GAM Computation Vehicle (mgcv) package [7] in the open source R statistical package [10] to estimate the model. The estimated probabilities of ignition were then used to simulate spatially and seasonally explicit ignitions for both natural and human caused fires. The estimated random year effects in the model for natural ignitions were not Gaussian. Consequently, in our simulations we generated a random year effect by sampling from the estimated empirical distribution.

Next we estimated a model for the probability of an ignition resulting in a fire greater than 10 ha. The model used ERC and spatial location as explanatories. We found no significant seasonal effect on the probability of an ignition becoming a large fire. Non-parametric smooth spline functions were included in the model to account for the effect of spatial location on the probability of ignitions becoming large fires. Fires >10 ha in our historical sample included 313 fires out of over 11,000.

Predicting fire size

The distribution of fire sizes, given an occurrence of a fire greater than 10 ha, was estimated using the log-generalized Pareto distribution with ERC as an explanatory variable [11]. We used a spline function for the relationship between the shape parameter and ERC, and a linear function for the relationship between the scale parameter and ERC.   For location and day with a simulated fire greater than 10 ha we used the estimated shape and scale parameters to simulate a fire size from the Pareto distribution.

Generating future ERC

The daily ERC values, which drive the estimation process, were derived from historical data from the RAWS climate archive referenced above. We used historical daily ERC values, averaged over the study area, to estimate an autoregressive model of order one with day-of-year

as an explanatory to account for seasonal patterns. The intercept is a random intercept to account for between year variability seen in the historical ERC data. This model was used to generate streams of 365 daily ERC values for each simulated year as specified by the user. Replicate fire lists are created by repeating the process creating a unique sequence of ERC values based on historical patterns.

Fuel moistures

Fuel moisture files are used by the FlamMap DLL to set moistures for each fuel size class (1-hr, 10-hr, 100-hr, 1000-hr) and fuel model [12] as well as live herbaceous and woody components. We used the historical (1987-2011) average fuel moisture values for each fuel class for each value of ERC used in the simulations.

Winds

Winds were modeled independently from fire probabilities. Wind direction was generated by randomly selecting from historical gust directions (1994-2011) from the Lave Butte RAWS station based on day-of-year. Wind speed based on gust was derived from the same weather data but was restricted to days in the historical record where fires occurred that exceeded 500 ha. Gust speed was sampled from a gust speed probability distribution generated from analysis of the Lava Butte RAWS data.

Calibrating burn periods

Because the MTT fire spread algorithm uses inputs on time rather than size of fire [13], simulating the fire predicted by the fire prediction system required a translation from fire size to burn period (minutes), and subsequent adjustments if the desired fire size was not achieved. In other words, it was necessary to guarantee that the simulated fire perimeters were materialized in any specific landscape with fire size and spatial distributions that reflect the predicted fire list by inputting a burn period and sampled historical weather. We accomplished this calibration by first generating a fire size – burn period distribution using FConstMTT, a command line version of FlamMap [13]. Specifically, we used 100 random ignition points and simulated wildfires with burn periods ranging from 30 min to 8000 min. Wind speed, azimuth and ERC were fixed at 18 mph, 220 degrees and 60, respectively. These simulated fire sizes and corresponding burn periods where used to predict burn period based on fire size with a second-order polynomial linear regression model. The model was applied to obtain an initial burn period for each predicted fire size on the fire list. We then tested the fire size prediction within FConstMTT simulations. We observed two issues with the approach, the first being that ignitions landed in non-burnable areas or in fuel limited situations, and the predicted fire size was not reached. To correct this first problem if the burn period and sampled weather conditions produced a simulated fire size smaller than the predicted fire size by a factor of 0.2, the ignition location (X, Y) was randomly sampled within a radius of 5 km, up to a maximum of five times. The X and Y coordinates of the simulated fire size that came closest to the predicted fire size replaced the original fire location. A second problem was observed where ignitions landed on fast burning

fuels and fire sizes were overestimated, or the sampled weather conditions were well above the mean conditions. In this case, if the fire size were more than 1.5 times the predicted fire size, the original burn period (BP) was reduced proportionally to the difference between the predicted and simulated fire sizes.

The resulting predicted versus simulated fire sizes were then plotted and examined for outliers and showed good correspondence although outliers weakened the relationship.

Fire prediction system

The above analyses were used to parametrize a fire prediction system written in R [10] that predicts daily fire occurrence and size from the model above and writes a text file fire list containing predicted fire weather parameters (wind speed and azimuth), burn probability, burn period, fire cause (natural or human) and fuel moisture conditions for each day a fire occurs in each simulation year. The fire lists are generated for a user-specified simulation period and number of replicates. We assessed the statistical model by using it to predict wildfire probability and size for 500 sample fires. The resulting data were compared to empirical data and both data sets plotted against ERC.

Historical ERC as determined from RAWS stations in the study area was related to both fire size and frequency. Increases in ERC corresponded to both more fires and larger fires. Results of simulated fire size distributions using the statistical model showed a similar pattern and distribution as the historical data. Thus we assumed the statistical model to be adequate for forecasting fire sizes from daily sequences of ERC. The statistical model to generate ERC streams for the fire prediction system generated a wide range of synthetic ERC streams that captured the daily and yearly variabilities in the observations as well as the seasonal pattern. The forecasted data also stayed approximately within the bounds of the observed data.

References

1. Finney MA, McHugh CW, Grenfell IC, Riley KL, Short KC. A simulation of probabilistic wildfire risk components for the continental United States. Stoch Env Res Ris A. 2011;25: 973–1000.
2. Preisler HK, Brillinger DR, Burgan RE, Benoit JW. Probability based models for estimating wildfire risk. Int J Wildland Fire. 2004;13: 133-42.
3. Short KC. A spatial database of wildfire in the United States, 1992-2011. Earth Syst Sci Data. 2014;6: 1-27. doi: 10.5194/essd-6-1-2014.
4. RAWS USA Climate Archive [Internet]. Western Regional Climate Center. 2014 [cited 1 October 2015]. Available from: http://www.raws.dri.edu/.
5. Bradshaw LS, Deeming JE, Burgan RE, Cohen JD. The 1978 National Fire-Danger Rating System: Technical documentation. Gen. Tech. Rep. Ogden, UT: USDA Forest Service, Intermountain Forest and Range Experiment Station, 1984 1983 July. Report No.: INT-169.

6. Preisler HK, Burgan RE, Eidenshink JC, Klaver JM, Klaver RW. Forecasting distributions of large federal-lands fires utilizing satellite and gridded weather information. Int J Wildland Fire. 2009;18: 508-16. doi: http://dx.doi.org/10.1071/WF08032.
7. Wood SN. Generalized Additive Models: An Introduction with R.: Chapman and Hall/CRC; 2006.
8. Brillinger DR, Preisler HK, Benoit J. Probabilistic risk assessment for wildfires. Environmetrics. 2006;17: 623–33.
9. Brillinger DR. Three environmental probabilistic risk problems. Statistical Science. 2003;18: 412-21.
10. R Core Team. R: A language and environment for statistical computing. 3.1.1 ed. Vienna, Austria: R Foundation for Statistical Computing; 2014.
11. Ager AA, Preisler HK, Arca B, Spano D, Salis M. Wildfire risk estimation in the Mediterranean area. Environmetrics. 2014;25(6): 384-96. doi: 10.1002/env.2269.
12. Scott JH, Burgan RE. Standard fire behavior fuel models: a comprehensive set for use with Rothermel's surface fire spread model. Gen. Tech. Rep. USDA Forest Service, Rocky Mountain Research Station, 2005 RMRS-GTR-153.
13. Finney MA. Fire growth using minimum travel time methods. Can J For Res. 2002;32(8): 1420-4. doi: 10.1139/x02-068.
