# Supplementary material for: Network analysis of wildfire transmission and implications for risk governance
Source: PLoS One. 2017 Mar 3;12(3):e0172867. doi: 10.1371/journal.pone.0172867 (PMC5336224; doi:10.1371/journal.pone.0172867)
Supplement: S1 Table — Fireshed area showing the area predicted to transmit fire into communities based on structure exposure with associated wildfire and structure exposure values. (PDF) [file pone.0172867.s002.pdf]

Table S1. Community fireshed area.

Community	Fireshed area (100 ha)	Fire received (ha yr^{-1})	Exposure (structures yr^{-1})	Fireshed capture [a] (i.e. specificity)	Exposure capture [b] (i.e. exposure specificity)	Federal land (%)	Federal land, protected [c] (%)
Beatty	505.8	93.31	1.28	0.91	0.79	0.27	0.25
Bend	592.7	172.60	198.2	0.92	0.66	0.42	0.35
Black Butte Ranch	105.4	9.55	12.58	0.76	0.48	0.91	0.58
Bly	293.1	36.47	1.48	0.88	0.76	0.47	0.00
Bonanza	540.8	48.69	0.82	0.86	0.42	0.24	0.00
Camp Sherman	85.1	4.50	3.65	0.48	0.54	0.96	0.92
Chemult	225.9	0.98	0.08	0.86	0.68	0.21	1.00
Chiloquin	410.1	16.94	3.34	0.9	0.82	0.43	0.83
Cline Falls	304.3	134.35	29.92	0.85	0.59	0.51	0.00
Cloverdale	303.4	47.57	4.63	0.82	0.55	0.53	0.14
Crane Mountain	89.5	3.44	0.02	0.5	0.18	0.79	0.24
Crater Lake West	7.5	0.09	0.01	0.25	0.38	1.00	1.00
Crescent	247.9	2.55	0.58	0.87	0.66	0.94	0.60
Dant	101.1	79.62	0.03	0.76	0.43	0.02	0.00
Drews Reservoir	23.1	0.31	0.02	0.37	0.23	0.97	0.00
Eagle Crest	248.2	80.45	34.97	0.88	0.55	0.38	0.00
Fort Klamath	518.7	15.34	0.11	0.88	0.63	0.53	0.58
Gateway	103.5	11.46	3.22	0.7	0.36	0.03	0.00
Gerber Reservoir	43.6	0.61	0	0.5	0.34	0.89	0.00
Gordon Lake	391.6	19.97	0.03	0.85	0.62	0.66	0.93
Horse Ridge	34.0	3.31	0.23	0.51	0.36	0.91	0.00
Keno	362.9	36.08	5.44	0.89	0.7	0.16	0.00
Klamath Falls	974.3	143.78	37.33	0.93	0.77	0.09	0.07
La Pine	555..6	55.92	26.42	0.93	0.84	0.65	0.39
Lake of the Woods South	70.6	1.13	0.03	0.52	0.28	0.83	0.35
Lakeview	801.9	106.73	5.61	0.93	0.83	0.35	0.08

Table S1. Continued.

Community	Fireshed area (100 ha)	Fire received (ha yr^{-1})	Exposure (structures yr^{-1})	Fireshed capture [a] (i.e. specificity)	Exposure capture [b] (i.e. exposure specificity)	Federal land (%)	Federal land, protected [c] (%)
Lorella	443.4	52.08	0.37	0.87	0.52	0.42	0.00
Malin	228.5	23.92	0.28	0.81	0.48	0.18	0.00
Merrill	374.2	17.09	0.47	0.91	0.66	0.17	0.00
New Pine Creek	212.8	17.29	0.3	0.8	0.52	0.31	0.41
Odell Lake	101.2	0.92	0.16	0.74	0.54	0.97	0.69
Paisley	353.0	22.03	0.21	0.87	0.78	0.61	0.16
Paradise Mountain	42.9	1.73	0.01	0.39	0.35	0.89	0.00
Pelican Bay	383.1	8.56	0.88	0.83	0.69	0.36	0.75
Pine Grove	88.8	2.44	0.1	0.55	0.27	0.04	0.00
Pine Mountain	66.9	0.82	0	0.67	0.56	0.92	0.27
Pronghorn	94.6	18.25	1.59	0.82	0.55	0.57	0.00
Quartz Valley	10.6	0.08	0.01	0.29	0.11	0.98	0.44
Redmond	205.0	23.25	26.41	0.77	0.51	0.25	0.00
Riverbed Butte Spring	130.3	3.87	0.05	0.88	0.72	0.72	0.37
Round Butte Dam	229.4	182.85	5.36	0.82	0.63	0.49	0.21
Sage Hen Butte	164.3	9.24	0.02	0.69	0.45	0.65	0.02
Silver Lake	588.8	22.20	0.22	0.86	0.59	0.73	0.01
Simnasho	96.7	7.08	1.11	0.63	0.4	0.00	0.00
Sisters	333.8	64.57	13.84	0.83	0.62	0.63	0.34
S'Ocholis Canyon	202.0	#N/A	0.77	0.81	0.47	0.73	1.00
Soloman Butte	493.6	41.51	0.14	0.91	0.66	0.37	0.35
Sprague River	657.6	110.08	2.6	0.92	0.77	0.37	0.88
Summer Lake	367.3	10.39	0.08	0.81	0.73	0.84	0.22
Sycan Marsh East	89.3	2.89	0	0.48	0.3	0.63	0.14
Three Rivers	264.3	46.15	58.08	0.93	0.63	0.69	0.52
Tumalo	351.9	59.80	12.31	0.81	0.39	0.39	0.21

Table S1. Continued.

Community	Fireshed area (100 ha)	Fire received (ha yr^{-1})	Exposure (structures yr^{-1})	Fireshed capture [a] (i.e. specificity)	Exposure capture [b] (i.e. exposure specificity)	Federal land (%)	Federal land, protected [c] (%)
Valley Falls	458.3	68.01	0.14	0.91	0.72	0.56	0.07
Warm Springs	280.9	504.65	36.83	0.87	0.59	0.01	0.00
West Side	560.2	72.76	0.77	0.88	0.51	0.32	0.01
Yonna	576.6	59.31	2.06	0.87	0.68	0.28	0.41

Fireshed area showing the area predicted to transmit fire into communities based on structure exposure with associated wildfire and structure exposure values.

[a] "Fireshed capture" = proportion of fires that lead to exposure that fell within the community fireshed boundary.
[b] "Exposure capture" = proportion of exposure (measured as structures) that came from fires within the fireshed boundary.
[c] Protected federal lands include national forest protected areas (e.g. conservation areas, wilderness) and Crater Lake National Park
